# Skin Immune Response of Immunocompetent and Immunosuppressed C57BL/6 Mice After Experimental Subcutaneous Infection Caused by *Purpureocillium lilacinum*

**DOI:** 10.3389/fmicb.2021.615383

**Published:** 2021-06-14

**Authors:** Danielly Corrêa-Moreira, Arethuza dos Santos, Rodrigo C. Menezes, Fernanda N. Morgado, Cintia M. Borba, Joseli Oliveira-Ferreira

**Affiliations:** ^1^Laboratory of Taxonomy, Biochemistry and Bioprospecting of Fungi, Oswaldo Cruz Institute, Oswaldo Cruz Foundation, Rio de Janeiro, Brazil; ^2^Laboratory of Immunoparasitology, Oswaldo Cruz Institute, Oswaldo Cruz Foundation, Rio de Janeiro, Brazil; ^3^Laboratory of Clinical Research in Dermatozoonosis in Domestic Animals, Evandro Chagas National Institute of Infectious Diseases, Oswaldo Cruz Foundation, Rio de Janeiro, Brazil

**Keywords:** *Purpureocillium lilacinum*, skin immune response, experimental model, immunosuppression, immunohistochemistry

## Abstract

Hyalohyphomycosis is a fungal infection characterized by the presence of a hyaline mycelium in the host. It is caused by several agents, such as *Purpureocillium lilacinum*. Our study aimed to evaluate some cell subsets and inflammatory markers involved in the *in situ* immune response to subcutaneous hyalohyphomycosis by *P. lilacinum* in C57BL/6 murine models. The fungal isolate was inoculated in mice randomly distributed in immunocompetent/infected (CI) and immunosuppressed/infected (SI) groups. Mice were evaluated on days 1, 3, 5, and 7 after inoculation. Histopathological studies showed several lesions in the site of infection as well as the formation of multifocal and mixed inflammatory infiltrates, which differed between the CI and SI groups. This analysis also revealed conidia and hypha-like structures in subcutaneous tissues of mice of both groups. The immunohistochemical analysis showed lower percentages of macrophages and neutrophils in the SI group compared to those in the CI group. Moreover, the intensity of interleukin (IL)-1β and nitric oxide synthase 2 production by cells of immunosuppressed mice was discreet, compared to immunocompetent mice that ranged from moderate to intense over time. The quantitative interference of dexamethasone in the response to the fungus was also demonstrated. We concluded that our results can be useful not only to broaden the knowledge on *P. lilacinum* but also, based on this host–parasite relationship, to contribute to the understanding of the mechanisms of infection.

## Introduction

Hyalohyphomycosis is a fungal infection characterized by the presence of a hyaline mycelium in the host (Ajello, 1986). It is caused by several agents, such as *Paecilomyces* spp., *Fusarium* spp., *Scedosporium* spp., *Scopulariopsis* spp., *Penicillium* spp., and *Purpureocillium lilacinum*. Some species, including *P. lilacinum*, in addition to hyaline mycelium have the ability to produce adventitious forms in the tissue, which are structures morphologically similar to phialides and microconidias that spread through the blood, causing occlusion, infarction, and vascular necrosis (Perfect and Schell, 1996; Das et al., 2010).

*Purpureocillium lilacinum* (Thom) Luangsa-ard, Houbraken, Hywel-Jones and Samson, comb. nov 2011, previously called *Paecilomyces lilacinus*, is a filamentous, hyaline, anamorphic fungus (Luangsa-Ard et al., 2011) considered as an emerging pathogen for humans, especially for immunosuppressed patients, although the number of immunocompetent hosts infected by this fungus has also increased in recent years (Shivaprasad et al., 2013; Turner and Conrad, 2015; Borba and Brito, 2016; Juyal et al., 2018).

Immunocompetent individuals present clinical manifestations that may vary from cutaneous to subcutaneous nodular lesions and constitute one third of the cases of hyalohyphomycosis by *P. lilacinum* (Pastor and Guarro, 2006). In immunosuppressed patients, the majority of reported cases were disseminated infections (Pastor and Guarro, 2006; Antas et al., 2011; Ding et al., 2014).

As the population of immunosuppressed individuals increases, fungal diseases have emerged as the major cause of human diseases (Shoham and Levitz, 2005; Antas et al., 2011). Patients with immune deficiencies frequently have recurrent mycoses and, in some cases, develop severe forms, which emphasize the importance of understanding how the immune system controls infection.

Although evaluations of immunological responses against *P. lilacinum* are scarce, it is generally believed that the main fungal defense mechanism is developed by phagocytes, which destroy fungi through the production of nitric oxide (NO) and other substances secreted by these cells (Romani, 2011). Usually, inflammatory infiltrates in cutaneous fungal infections are composed of lymphocytes, neutrophils, and macrophages. Granulomatous reactions, often suppurative, are observed, depending on the immunological status of the patient (Guarner and Brandt, 2011).

Miranda et al. (2016) evaluated cats naturally infected with *Sporothrix* spp. and demonstrated that the fungus stimulates the development of granulomas with phagocytes that are unable to destroy fungal cells and that the inhibition of macrophage functions increased host susceptibility. These results corroborate the data of Antachopoulos and Roilides (2005), who mentioned the participation of granulocyte colony-stimulating factor (G-CSF), macrophage colony-stimulating factor (M-CSF), and granulocyte-macrophage colony-stimulating factor (GM-CSF), important glycoproteins in the production and activation of phagocytes that are crucial in the defense against fungi. Previously, Sa et al. (2007) also demonstrated the presence of Langerhans cells in the cutaneous tissue of patients with chromoblastomycosis, pointing out that the persistence of the fungus in the tissue is related to the activation of macrophages and the production of NO.

In patients with cutaneous forms of hyalohyphomycosis caused by *P. lilacinum*, several authors describe the presence of mixed inflammatory infiltrates in the lesions, composed of macrophages, neutrophils, and dendritic cells (DCs) (Gutierrez-Rodero et al., 1999; Lin et al., 2008; Huang et al., 2011). Since cutaneous infections constitute the majority of cases of fungal hyalohyphomycosis, it is necessary to emphasize the importance of these cells in the phagocytosis and presentation of antigens and, consequently, in the efficiency of the adaptive response (Antachopoulos and Roilides, 2005).

The impact of immunosuppression by glucocorticoids on the immune response is due to their mechanisms, which promote the inhibitory effect of the synthesis of pro-inflammatory cytokines such as interleukin (IL)-2, IL-6, and tumor necrosis factor (TNF)-α, and prostaglandins (Song et al., 2005). According to Lionakis and Kontoyiannis (2003), glucocorticoids affect the number of mononuclear leukocytes, causing reversible lymphopenia and monocytopenia. The drug primarily affects cellular immunity, both qualitatively and functionally, depleting circulating CD4^++^ T lymphocytes, and to a lesser extent CD8^+^ T lymphocytes, in addition to monocytes, macrophages, and polymorphonuclear cells. In the murine model of systemic infection, de Sequeira et al. (2017) demonstrated that immunosuppressive drugs such as dexamethasone interfere with lymphoproliferative responses. However, the authors did not evaluate the influence of the drug on subcutaneous infection.

Thus, this study aimed to evaluate some cell subsets and inflammatory markers involved in the skin immune response in subcutaneous hyalohyphomycosis by *P. lilacinum* due to its emerging and opportunistic potential in an immunocompetent and immunosuppressed murine model, characterizing phenotypically and functionally some cells involved in this process.

## Materials and Methods

### Ethics Statement

This study was performed in strict accordance with the Brazilian College of Animal Experimentation (COBEA). All experimental procedures involving animals followed the regulations for animal experiments of the Ethics Committee for Animal Study of FIOCRUZ (license number L-031/2015-CEUA/IOC-FIOCRUZ) and were performed under anesthesia. All efforts were made to minimize the suffering of the animals.

### Mice

Ninety-six male C57BL/6 mice, aged 6–8 weeks, weighing approximately 21 g were used in the experiments. The number of animals used in this study was calculated to guarantee statistically valid results obtained with as few individuals as possible without compromising the degree of reliability and avoiding the unnecessary use of animals.

Mice were randomly allocated to each experimental group and were kept under specific pathogen-free conditions at the Laboratory of the Animal Experimentation Center (IOC FIOCRUZ) and maintained in micro-insulators made of polypropylene, autoclavable, containing pine beds. Micro-insulators were disposed in ventilated racks (ALESCO) with air exchange measures of 350 m^3^/h. Breeding room was managed at room temperature; 23–25°C, humidity; 30–70%, light/dark cycle; 12 h, and water and food were given *ad libitum*. We took the utmost care to alleviate any pain and suffering on the part of the mice. Mice were submitted to euthanasia by CO_2_ exposure prior to analysis.

### Purpureocillium lilacinum

One human strain of *P. lilacinum* was used in this study: S2, kindly provided by Dr. Annette Fothergill (Fungus Testing Laboratory, University of Texas Health Science Center–San Antonio, San Antonio, TX, United States), isolated from skin biopsy on the left foot of a female patient. The strain was molecularly authenticated and deposited in GenBank^®^ sequences database with access number MF590109 (de Sequeira et al., 2017).

### Culture Conditions and Inoculum

The isolate was subcultured on potato dextrose agar (PDA) medium at room temperature for 12 days, and then, conidia were collected by scraping the colonies, suspended in phosphate buffered saline (PBS), followed by thermal shock protocol to separate conidia from hyphae, according to de Sequeira et al. (2017). The supernatant was collected for quantification in a Neubauer hemocytometer. Cell viability was assessed by colony-forming unit (CFU) (Goihman-Yahr et al., 1980). The resulting suspensions were adjusted to the desired inoculum.

### Immunosuppression by Dexamethasone

To perform immunosuppression, mice received 1 mg/kg of dexamethasone (Hypofarma, Ribeirão das Neves, Brazil) administered *ad libitum* in the drinking water for 3 days before fungal inoculation and during all the experiments (Brito et al., 2011). We chose dexamethasone because of its ability to deplete CD4^+^ T lymphocytes, similar to the condition provoked by HIV infection, one of the preexisting factors for fungal infections. Tetracycline (1,000 mg/L; Teuto, São Paulo, Brazil) was also added to the drinking water in parallel to prevent bacterial infections. The route of administration of the drug had already been demonstrated by our group as safe and effective in inducing immunosuppression without, however, causing stress in animals as do invasive methods like gavage (Brito et al., 2011; de Sequeira et al., 2013, 2017).

### Experimental Hyalohyphomycosis

The fungal isolate was inoculated subcutaneously by a syringe at the base of the tail in 32 mice randomly distributed into two groups: immunocompetent (CI) and immunosuppressed mice (SI). Sixteen mice were distributed into immunocompetent (CC) and immunosuppressed mice (SC) control groups and similarly inoculated with PBS. In dorsal–ventral position, mice were inserted in an acrylic container, and after trichotomy and asepsis of the tail base region, the CI and SI groups were inoculated with 0.02 ml of the cell suspension containing 1 × 10^5^ conidia of *P. lilacinum* with more than 80% viability. Control groups (CC and SC) were similarly inoculated with PBS. Mice were checked daily for 7 days to observe abnormal behaviors and pathological changes, with the aim of minimizing suffering. On each observation point (days 1, 3, 5, and 7 after inoculation), four animals from the CI and SI groups and two from the CC and SC groups were euthanized.

### Histopathology

Mice were submitted to anesthesia with 10% ketamine hydrochloride associated with 2% xylazine hydrochloride (Syntec, Santana de Parnaíba, Brazil) and submitted to euthanasia by CO_2_ exposure. After asepsis of the region, two skin fragments of approximately 3 mm in diameter around the point of inoculation were removed. One skin fragment was submitted to histological analysis and the other to immunohistochemistry.

The skin fragment destined for histopathology was fixed in 10% neutral buffered formalin, dehydrated, and embedded in paraffin. Sections were cut and stained with hematoxylin–eosin (H&E), periodic acid-Schiff (PAS), and Grocott (methenamine silver), and the photographs were taken with a Leica DM 1000 (Leica, Werzlar, Germany). The inflammatory infiltrate in tissues was classified into: granulomatous, predominance of macrophages, and non-granulomatous, predominance of other types of inflammatory cells (such as lymphocytes, plasma cells, and neutrophils). The intensity of the inflammatory infiltrate was classified as follows: absent or mild (cellular infiltrate absent or mild and dispersed foci) and moderate to intense (cellular infiltrate dense and diffuse).

### Immunohistochemistry

Biopsy fragments frozen in OCT resin (Sakura Finetek, Alphen aan den Rijn, Netherlands) were cut into 3-μm-thick sections and mounted on microscope silanized slides (Dako Cytomation, Carpinteria, CA, United States). The slides were fixed in acetone PA (Merck, Darmstadt, Germany) and hydrated in PBS, pH 7.4. Blockage of endogenous peroxidase (peroxidase blocking reagent; Dako, Carpinteria, CA, United States) and non-specific staining (normal goat serum; Zymed Laboratories Inc., San Francisco, CA, United States) were performed before the use of specific antibodies.

The specimens were then incubated with the primary antibodies directed to surface receptors: CD68^+^ macrophages, anti-neutrophilic elastase, nitric oxide synthase type 2 (NOS2), and IL-1β (all from BD Biosciences Pharmingen, San Diego, CA, United States), followed by incubation with the biotinylated secondary antibody (Zymed) and the streptavidin–biotin–peroxidase complex (ABC kit; Dako Cytomation). Aminoethylcarbazole (AEC kit; Zymed) was used as the substrate–chromogen system, and the slides were counterstained with Mayer’s hematoxylin (Dako). The slides were examined under a light microscope (E400 model; Nikon Instruments Inc., Melville, NY, United States), and the percentage of stained cells was determined by counting 500 cells as standard. The intensity of IL-1β and NOS2 was scored in 10 microscope fields. + = discreet (at least one positive site per field); ++ = moderate (two or three positive sites per field); +++ = intense (four or five positive sites per field).

### Statistical Analysis

Data were analyzed using GraphPad Prism R software, version 8 (GraphPad Software, Inc., San Diego, CA, United States). The Kolmogorov–Smirnov test was used to evaluate the distribution of variables. Mann–Whitney test was used to perform comparisons between groups. Data are expressed as mean ± standard deviation. The *p*-value cutoff for statistical significance was 0.05, and the n value for each group on each time point = 4.

All the experiments were carried out twice to ensure reproducibility.

## Results

### Clinical Signs

**FIGURE 1:**
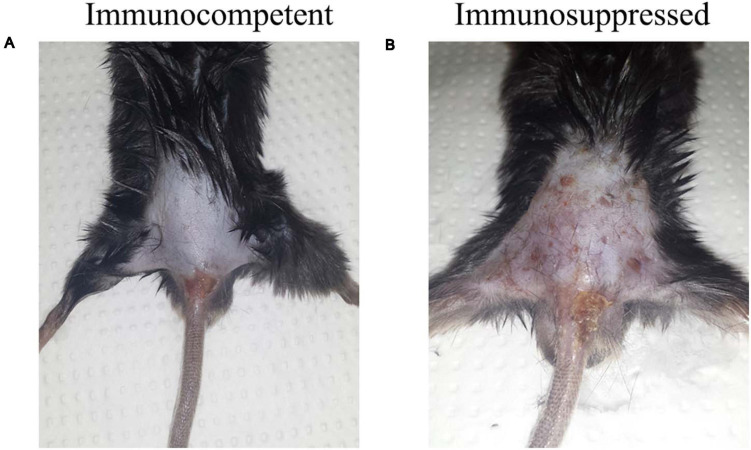
Macroscopic aspects of the epidermis of the immunocompetent (CI) and immunosuppressed (SI) mice inoculated at the tail base with 1 × 10^5^ conidia of *Purpureocillium lilacinum* on day 5 after infection. **(A)** Discreet, non-ulcerative, and non-suppurative lesion at the point of inoculation; **(B)** Crusted lesion at the point of inoculation, extending to the base of the tail.

Mice were observed for 7 days. No clinical alterations as apathy and weight and/or hair loss were observed in the immunocompetent (CI/CC) group. The inflammatory response at the site of the infection was discreet, non-suppurative, and non-ulcerative (Figure 1A). On the other hand, mice in the immunosuppressed group (SI/SC) presented apathy and hair loss and crusted lesions at the point of inoculation, extending to the base of the tail (Figure 1B).

### Histological Studies

**FIGURE 2:**
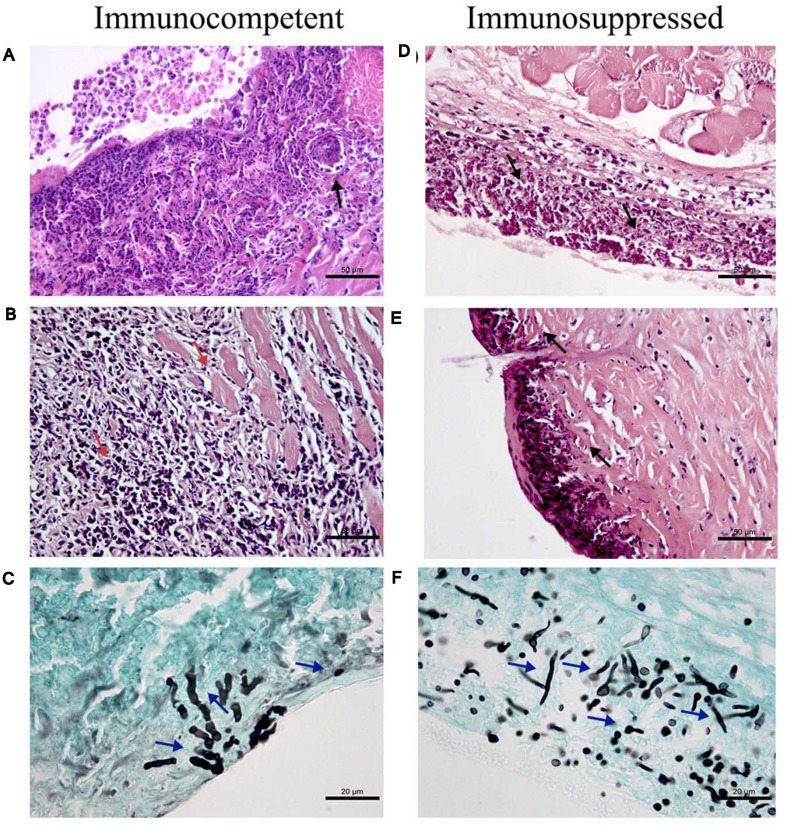
Histological findings in the cutaneous and subcutaneous tissues of immunocompetent (CI–left column) and immunosuppressed mice (SI–right column) inoculated at the tail base with 1 × 10^5^ conidia of *Purpureocillium lilacinum*. **(A)** Diffuse inflammatory infiltrate (black arrow) constituted mainly by macrophages and neutrophils reaching superficial and deep dermis on day 1 after infection, H&E. **(B)** Necrosis in the skeletal muscle and fibrosis in the dermis and subcutaneous tissue (red arrows) on day 5 after infection, H&E. **(C)** Hyphae in the dermis (blue arrows) on the third day after infection, Grocott. **(D)** Severe inflammatory infiltrate (black arrows) affecting the superficial and deep dermis and adjacent skeletal muscles on day 3 after infection, H&E. **(E)** Suppurative dermatitis and panniculitis (black arrows) with diffuse inflammatory infiltrate ranging from mild to severe consisting mainly of neutrophils, with fewer macrophages on day 7 after infection, H&E; **(F)** Abundant hyphae, pseudohyphae (blue arrows) in the subcutaneous tissue on day 1 after inoculation, Grocott.

Figure 2 shows histological findings in the cutaneous and subcutaneous tissues of immunocompetent and immunosuppressed mice inoculated at the tail base with *P. lilacinum*.

Immunocompetent mice inoculated with *P. lilacinum* (CI group) presented dermatitis, panniculitis, and skin ulcerations with diffuse inflammatory infiltrate reaching superficial and deep dermis (Figure 2A–day 1), consisting mainly of neutrophils and macrophages in all days during the follow-up (1, 3, 5, and 7 days) after inoculation. From the third day after the inoculation, hyphae were detected, as well as abundant intracellular and extracellular conidia (Figure 2C), and on day 5, necrosis of the skeletal muscle and foci of fibrosis in the dermis and subcutaneous tissue were observed (Figure 2B). Conidia within macrophages and neutrophils were observed in the subcutaneous tissue and also in the superficial dermis in the middle of the inflammatory infiltrate. On day 7 after inoculation, the skin of these mice presented acanthosis, and there were conidia inside the macrophages (data not shown).

In general, immunosuppressed mice inoculated with *P. lilacinum* (Group SI) had more severe lesions and more fungal structures than the immunocompetent ones (CI Group) from the first day after inoculation. In all the sampling days, the histological analysis of the tissue collected from these animals revealed the formation of diffuse and/or multifocal inflammatory infiltrate, which intensified until the end of the experiment. Extensive areas of ulcer covered by crusts, dermatitis, and suppurative panniculitis were also observed (data not shown).

On day 1, similarly to the immunocompetent group, numerous conidia in the interior of macrophages and hyphae in the tissue were revealed (Figure 2F). On the third day after inoculation, the infiltrate ranged from moderate to severe, and the extension of the infiltrate affected the superficial and deep dermis and adjacent skeletal muscles (suppurative myositis), being more intense in the subcutaneous tissue (Figure 2D). Five days after inoculation, in addition to the presence of conidia inside the phagocytes and in the tissue, hyphae were also observed in the crusts that covered the ulcerations in the epidermis of immunosuppressed mice (data not shown), also observed at the end of the experiment (day 7), with the formation of multifocal inflammatory infiltrates, besides abundant conidia and hyphae in the crusts, dermis, and mainly subcutaneous tissue (Figure 2E). In all samples (CI and SI groups–1, 3, 5, and 7 days after infection), the culture was positive to *P. lilacinum*. As expected, all control mice (Groups CC and SC) were negative.

### Immunohistochemistry

**FIGURE 3:**
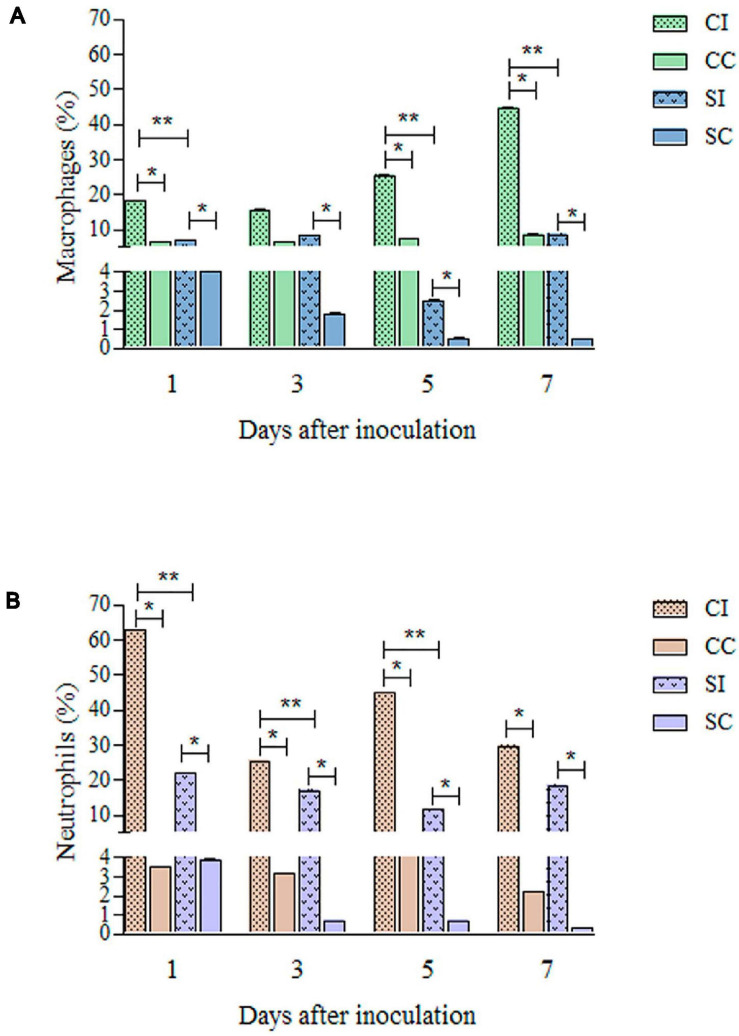
Quantification of macrophages **(A)** and neutrophils **(B)** from skin biopsy of the tail base region of immunocompetent and immunosuppressed mice inoculated with *Purpureocillium lilacinum* and control groups inoculated with phosphate-buffered saline (PBS) by immunohistochemistry. CI, Immunocompetent mice inoculated with *P. lilacinum*; CC, Immunocompetent mice inoculated with PBS; SI, Immunosuppressed mice inoculated with *P. lilacinum*; SC, Immunosuppressed mice inoculated with PBS. *Statistical differences between infected and control groups (*p* < 0.05). **Statistical differences between immunocompetent and immunosuppressed groups (*p* < 0.005).

Figure 3 shows the differences in macrophage and neutrophil frequencies and the intensity of inflammation markers, comparing immunocompetent and immunosuppressed groups. It was possible to observe lower percentages of macrophages and neutrophils in the SI group, compared to CI, as well as a lower intensity of production of IL-1β and NO by the cells of immunosuppressed mice than by those of immunocompetent ones.

**FIGURE 4:**
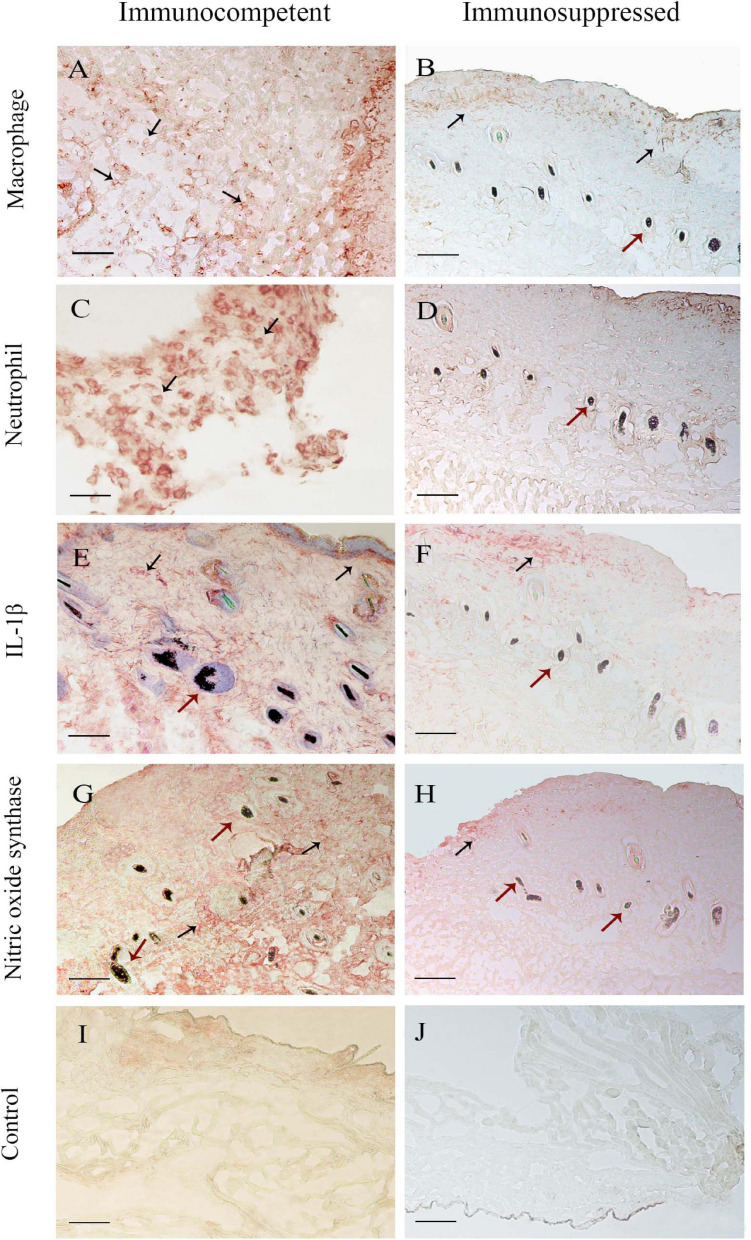
Immunohistochemical analysis of skin biopsy of immunocompetent (left column) and immunosuppressed (right column) mice 7 days after infection. Mice were inoculated at the tail base with 1 × 10^5^ conidia of *Purpureocillium lilacinum* and control groups were inoculated with phosphate-buffered saline (PBS). The black arrows show the frequencies of **(A,B)** macrophages and **(C,D)** neutrophils and the intensity of interleukin (IL)-1β **(E,F)** and nitric oxide synthase **(G,H)** production. **(I,J)** Control group. The red arrows signal the hair follicle of mice tail epidermis (— = 100 μm).

The immunohistochemical analyses during the follow-up are summarized in Figure 4. In the immunocompetent group infected with *P. lilacinum* (CI group), the percentages of macrophages (Figure 4A) were lower than those of neutrophils (Figure 4C), especially on day 1 after inoculation. However, concerning the quantification of macrophages, it was possible to notice that the absolute number of these cells tended to increase in the final observation points, as well as the expression of NOS2. IL-1β (Figure 4E) production was high on the first day after infection, decreased on days 3 and 5, and showed an elevation at the end of the experiment. The production of NOS2 (Figure 4G) was moderate on days 1 and 3, increasing from day 5 until the end of the experiment. Compared to the infected group (CI), the production of IL-1β and NOS2 in the control group (CC) was discreet over time.

It was not possible to reach 500 cells in the immunosuppressed mice of both infected (SI) and control (SC) groups. Approximately 280 cells were quantified in these animals and, as observed in immunocompetent mice, there was a higher percentage of neutrophils than macrophages (Figures 4B,D), especially in the initial (day 1) and final (day 7) periods of the experiment. The production of NO and IL-1β remained discreet (Figures 4F,H), without variations over time, in the SI and SC groups.

## Discussion

Currently, invasive fungal infections are one of the greatest threats for immunosuppressed hosts (Antachopoulos and Roilides, 2005). Hyalohyphomycosis is caused by several agents, and *P. lilacinum* (Antas et al., 2011) is one of them.

The mechanisms of cellular and immunological response against this fungus are scarce; therefore, this work sought to investigate part of the basis of the host–parasite interaction. Previous studies of our group using peritoneal macrophages from C57BL/6 mice lineage for *in vitro* experiments demonstrated the potential of this fungus to adhere, invade, and destroy host cells (Peixoto et al., 2010). Since these results pointed out the possibility of disease establishment in this mouse strain, we chose the *in vivo* C57BL/6 murine model with the same genetic lineage of the cells previously used.

Notwithstanding *P. lilacinum* fungus has low virulence, according to some authors, and requires an immunosuppressed host to invade and produce the disease (Hubálek and Hornich, 1977; Pujol et al., 2002; Pastor and Guarro, 2006), both immunocompetent and immunosuppressed models used by our group were infected, although the immunosuppressed model seemed to be more susceptible.

The ability of the fungus to escape the immune response of the host and remain viable and circulating was demonstrated by de Sequeira et al. (2017). In this work were considered mechanisms of the adaptive immune response, as the role of CD4^+^, CD8^+^, regulatory T cells (Tregs), and memory T cells, of immunocompetent and immunosuppressed mice infected with *P. lilacinum* for a period of up to 45 days. It was possible to observe the impact of dexamethasone in the course of the infection, as presented here, allowing us to observe that the administration of dexamethasone can quickly (within 2 h) modify the immune response (Bain et al., 2018).

The study of de Sequeira et al. (2017) also reports that immunocompetent animals, inoculated intravenously, remained healthy during the experimental period and did not present any behavioral changes or clinical signs, such as weight loss and internal or external organ damage. However, the infection was established, since viable fungal cells were recovered from the spleen. Moreover, conidia-like structures were found in the histopathological analysis of the lung and liver of the mice of this group. In contrast, immunosuppressed mice of the same study showed weight loss, hair loss, apathy, dermatitis, internal organ damage, and keratitis, consistent with the reported cases of human infection and similar to the BALB/c model used by Brito et al. (2011).

In our study, we used the same mouse model as that of de Sequeira et al. (2017), but the route of inoculation was subcutaneous in order to evaluate the early stages of *P. lilacinum* cutaneous infection. Our results showed similar clinical signs to those of animals inoculated intravenously (Brito et al., 2011; de Sequeira et al., 2017). The immunocompetent mice did not present any clinical signs, while the immunosuppressed group presented apathy and hair loss. However, both groups presented lesions at the site of infection, dermatitis, and in some cases, keratitis.

The results of this work go in line with those obtained by Permi et al. (2011); Saghrouni et al. (2013), and Bassiri-Jahromi (2014), whose histopathological analysis of the skin biopsies of immunocompetent and immunosuppressed patients infected with *P. lilacinum* revealed the formation of granulomatous reactions, in which inflammatory infiltrates are mainly composed of polymorphonuclear and macrophages. We also observed ulcerated lesions at the site of infection in mice, as well as the formation of multifocal and mixed inflammatory infiltrates, that differed between immunocompetent (CI) and immunosuppressed (SI) groups with respect to severity—more pronounced in SI. It is interesting to highlight that in the study published by Permi et al. (2011), reporting the infection caused by this fungus in an immunocompetent patient, the formation of fibrosis was observed, and the same was shown in our immunocompetent murine model. It is known that fibrosis is the formation of tissue composed of collagen fibers in an organ in response to injury or tissue damage, so we speculate that the normal host immune condition uses this mechanism to respond to the invasion and proliferation of the fungus in host tissues.

The immunohistochemical analysis of the tissue fragments collected from the immunocompetent (CI and CC) and immunosuppressed (SI and SC) mice allowed us to identify and quantify macrophages, neutrophils, and two inflammatory markers, IL-1β and NOS2, involved in the immune response *in situ*. In general, percentages of neutrophils in immunocompetent and immunosuppressed mice were higher than those of macrophages, except for the seventh day after inoculation, in the immunocompetent group. We know that *P. lilacinum* is capable of destroying macrophages that phagocytose the conidia, as well as forming germ tubes and hyphae in the first 24 h after infection *in vitro* (Peixoto et al., 2010). Thus, the marked difference in the percentages of these cells can be attributed to the fact that, although macrophages are the first line of defense against conidia, neutrophils are the cell population selected to eliminate hyphae (Schaffner et al., 1982; Cenci et al., 1997). This hypothesis is plausible to assume, since *P. lilacinum*, as well as other agents of hyalohyphomycosis, is a producer of hyphae and adventitious structures in the tissue (Perfect and Schell, 1996). These results confirm the findings of Demirezen et al. (2015), since these authors verified the predominance of neutrophils compared to macrophages in the cervicovaginal smear of patients infected with *Candida* spp., which are able to produce hypha and/or pseudohypha.

In contrast, Morgado et al. (2011) observed higher percentages of macrophages compared to neutrophils in the fixed and lymphocutaneous forms of sporotrichosis. We may suppose that these results are due to the fact that *Sporothrix* spp. in the host acquire the yeast form against which the macrophage-mediated immune response may be effective. The difference in the number of macrophages found in this study between the immunocompetent and immunosuppressed groups is also worth noting, demonstrating once again the action of dexamethasone. We know that the drug, by interfering with IL-2 synthesis, can suppress the production of inflammatory markers, such as IL-1β (Paliogianni et al., 1993; Antachopoulos and Roilides, 2005; Orlikowsky et al., 2005), leading to a deficiency in macrophage function. Our results corroborate these data, since the production of NOS2 and IL-1β by immunocompetent mice ranged from moderate to severe, especially in the final periods of observation, while in immunosuppressed mice, it remained discreet on all points.

In conclusion, our data reinforce that host conditions are crucial to the severity of opportunistic infection by *P. lilacinum*, since it is capable to infect both immunocompetent and immunosuppressed murine models but causes damage only in the immunosuppressed ones. The lower percentages of innate immune cells and their products in immunosuppressed mice demonstrate the quantitative interference of dexamethasone in the response to the fungus. Thus, we conclude that our results have not only broadened the knowledge on *P. lilacinum* but also, based on this host–parasite relationship, contributed to the understanding of the mechanisms of infection.

## Data Availability Statement

The original contributions presented in the study are included in the article/supplementary material, further inquiries can be directed to the corresponding author/s.

## Ethics Statement

The animal study was reviewed and approved by Ethics Committee for Animal Study of FIOCRUZ (license number L-031/2015- CEUA/IOC-FIOCRUZ).

## Author Contributions

DC-M, JO-F, and CB designed the study. DC-M and AS carried out the experiments. DC-M, RM, and FM analyzed the data. DC-M wrote the manuscript. DC-M, RM, FM, CB, and JO-F revised and approved the final manuscript. All authors contributed to the article and approved the submitted version.

## Conflict of Interest

The authors declare that the research was conducted in the absence of any commercial or financial relationships that could be construed as a potential conflict of interest.
